# A Short Note About the Reconsideration of the Image of Bayezid II Darüşşifa

**DOI:** 10.4274/balkanmedj.2017.3.0001

**Published:** 2017-05-15

**Authors:** Öner Kıranlar, Zafer Koçak

**Affiliations:** 1 Department of Sculpture, Trakya University School of Fine Arts, Edirne, Turkey; 2 Department of Radiation Oncology, Trakya University School of Medicine, Edirne, Turkey

Sultan Bayezid II Külliyesi was constructed by Architect Hayrettin between 1484 and 1488. Alongside the Mosque, Külliye (complex) includes primarily Darüşşifa (hospital), Madrasah (university), Tabhane, İmaret (poorhouse), Bathhouse, Treadmill, Bridge, Mehterhane, Sıbyan Mektebi and Muvakkithane. The foundation of Sultan Bayezid II Külliyesi has been a key point both in Ottoman architecture and in the social and political transformation of Ottoman State. This key point was a specific historical period when Ottoman State took the scientific and political leadership of the Islamic World and reorganized its structure by this fact. One can say that the infrastructure of the transformation of Devlet (State) into an empire was constituted in this period. The philosophical, religious and scientific heritage that is developed within the zawiyahs of Horasan, North Syria and Iraq had been a vanguard of great cultural leap merging with the ancient world represented by The Asklepion of Pergamon. The Ottoman medicine which was founded on the heritage of great medicines and philosophers like Avicenna Abū Rayḥān Al-Bīrūnī, Al-Farabi, Razi, Abū al-Qāsim al-Zahrāwī, and Maimonides embodied in Darüşşifa and Medicine Madrasah in the Islamic and Turkish world (1). Generally built within the Külliye complexes, these multifunctional structures were places of new researches in which theoretical and practical aspects of medicine had been performed (2). The privileged position of medicinal education within the Ottoman education system raised the scientific quality of the researches within these institutions above all its counterparts. Evliya Çelebi tells this sophistication and richness as follows: “In the rooms of Medrese-i Etibba (medicine school), there are disciples who are dignified medicines, always speaking of savants like Platon, Socrates, Philebos, Aristotle, Calinos, Pythagoras. Each has chosen a discipline and referring to valuable volumes of the medicinal science, endeavors to find a cure for the mankind” (3). This versatility provided experimental practices like music and occupation therapy, alongside the traditional medicinal practices of the period (4,5,6). The Fall of Ottoman Empire had also been the fall of these institutions and they had been abandoned to their fates. In 1984, the restoration process had been undertaken by Trakya University and by 1997 the buildings were transformed into the medicine and health museum. Since that day, the new function of structure has been the presentation of the past. This new function may also be called as the proper constitution of the bond between the past and present and carrying it to future.

The science has a cumulative structure. The new advances are possible only as results of present conditions. In our days, the qualities of the institutions conducting scientific researches have not only been determined by their technological possibilities but also by their historical and cultural potentialities. Thus, many prestigious scientific institutions embrace their history institutionally through medieval monasteries and traditionally through the ancient lore. “BALKAN MEDICAL JOURNAL” of School of Medicine of Trakya University, symbolizing its historical heritage with the silhouette of Bayezid II Şifahanesi on its new logo and name during this new period, constitutes a visual memory of the transition of tradition to the future by a bold and innovative interpretation of tiling colors.
